# Work Readiness and Vocational Belonging: Exploring the Experiences of Newly Graduated Nurses

**DOI:** 10.1002/nop2.70404

**Published:** 2026-01-07

**Authors:** Berra Yilmaz Kusakli, Ece Uysal Kasap

**Affiliations:** ^1^ Department of Nursing, Faculty of Health Sciences Istanbul Aydin University Istanbul Turkiye; ^2^ Istanbul Provincial Health Directorate Istanbul Turkiye

**Keywords:** newly graduated nurse, nursing, vocational belonging, work readiness

## Abstract

**Introduction:**

This study aims to explore the relationship between work readiness and vocational belonging among newly graduated nurses‐(NGNs).

**Methods:**

The study employed a descriptive, correlational, and cross‐sectional design. It was conducted with 415 NGNs working at a city hospital in Istanbul, Türkiye. The Work Readiness Scale‐Newly Graduated Nurses‐(WRS‐NGN) and the Vocational Belonging Scale‐(VBS) were used for data collection. The data obtained from the study were analysed using the SPSS.22.0 statistical software. For data analysis, descriptive statistics such as frequency, percentage, and mean were calculated, and inferential statistics including *t*‐tests, ANOVA, Pearson correlation, and regression analysis were performed.

**Results:**

The mean WRS‐NGN score of the participants was 347.29 ± 55.37, while the mean VBS score was 121.81 ± 35.45. A positive and moderate correlation was found between the work readiness and vocational belonging levels of NGNs (*r* = 0.607, *p* < 0.01).

**Conclusions:**

Work readiness was found to explain 36.8% of the variance in vocational belonging levels. The findings of the study revealed that NGNs were well‐prepared for work and exhibited a moderate level of vocational belonging. It was also determined that the work readiness of NGNs influenced their level of vocational belonging.

**Patient or Public Contribution:**

No Patient or Public Contribution.

## 1 Introduction

As nursing students approach the end of their education, they acquire the necessary legal and professional qualifications to enter the workforce as new graduates. This transition represents a shift from their role as students to that of professional nurses, integrating into the healthcare system (Baris et al. 2024). The healthcare system is dynamic and multifaceted, characterised by rapid advancements in medical technologies, increasing patient acuity, and a critical shortage of nursing staff (Yildiz Keskin and Aslan 2021). Within this context, newly graduated nurses (NGNs) often perceive the transition into the workforce as highly challenging, navigating complex individual, interpersonal, clinical, and organisational dynamics (Hallaran et al. 2022). NGNs who report difficulties in meeting the escalating demands of the profession often express that these challenges have significant physical, emotional, and personal impacts (Siew Hoon et al. 2024). Consequently, the first year of professional practice is frequently perceived as a critical adjustment period and is a key determinant in decisions regarding job retention and career longevity within the nursing profession (Qu et al. 2025). This situation may arise from a disconnect between the theoretical foundations of nursing education and the practical realities of the profession, alongside insufficient preparation for the demands of the workplace and low levels of vocational belonging (Kox et al. 2020; Punt 2022). In this context, the aim of this study was to explore the relationship between work readiness and vocational belonging among NGNs.

## 2 Background

A specific period of time is recognised as crucial for the development of professional skills and competencies among NGNs (Charette et al. 2020). This period is generally understood to fall within the first 3 years following the receipt of their nursing licence (Masso et al. 2022). According to Benner (1984), nurses progress through various stages of professional development, from novice and advancing to advanced beginner, competent, proficient, and expert. This model can help explain how work readiness and vocational belonging develop as newly graduated nurses gain knowledge and experience. New graduates begin as novices with limited experiential knowledge, creating a gap between academic preparation and workplace demands. This transition period is critical for developing work readiness and establishing professional identity. As new nurses gain experience and expertise, they can perform their jobs more effectively and efficiently, and at the same time feel a stronger sense of professional identity (Benner 1984).

During this transition, understanding the work readiness of nurses is critical to comprehending how NGNs navigate various levels of competence and refine their clinical expertise (Siew Hoon et al. 2024). Work readiness theory concedes that transition to professional practice is multifaceted, and to be successful graduates must attain multiple capabilities and characteristics Work readiness encompasses four dimensions of professional attributes and skills for graduate nurses that extend beyond academic ability: organisational acumen, social intelligence, personal work characteristics and work competence (Walker et al. 2015). Generic skills such as effective communication, problem solving and resilience are categorised within these domains to provide a systematic framework for improved understanding of transition success (Caballero et al. 2011). Work readiness in nursing refers to the attitudes, skills, and characteristics necessary for successful clinical practice (Brown et al. 2022; Kim and Shin 2022). It encompasses multidimensional knowledge and skills that enable them to deliver safe, high‐quality care, and work independently. These types of competencies are developed through systematic education provided to NGNs by universities and healthcare institutions (Kim and Shin 2022). However, the COVID‐19 pandemic, which has persisted for nearly two years, has significantly impacted both the healthcare and education systems (Baris et al. 2024; UNDP 2020). This sudden situation introduced unprecedented challenges, especially in the field of healthcare education (Dentice et al. 2024). As a result of the rapid global proliferation of COVID‐19, on March 13, 2020, Türkiye, like many other nations, decided to suspend in‐person instruction at schools and universities and transition to distance learning methodologies (UNDP 2020; UNE 2020). Among other reasons, the uncertainty regarding the severity of the disease, the lack of the personal protective equipment available and the likelihood of contracting COVID‐19 or becoming vehicles of transmission within health‐care facilities (AAMC 2020) imposed the interruption of the traditional nursing education in several countries. A new approach to education became inevitable transforming mandatory face‐to‐face teaching to distance learning whit digital learning (Dewart et al. 2020). Several studies have investigated the effectiveness of e‐learning, its advantages and disadvantages (insufficient digital competencies and equipment), developing a body of evidence useful to design changes in the post‐restrictions era (Dentice et al. 2024). In this context Nurses who trained during the pandemic graduated with less real‐world experience due to clinical internship restrictions. This led to new graduates feeling less prepared when they started their jobs and a longer adaptation period (Dewart et al. 2020). In addition, these newly graduated nurses started to work mainly during the Covid‐19 Pandemic.

The concept of work readiness is closely linked to the retention of NGNs within the healthcare workforce and the nursing profession (Hallaran et al. 2022; Qu et al. 2025), serving as a key indicator of career potential, performance, and opportunities for advancement (Ma et al. 2021). Masso et al. (2022) review of 45 studies examining the readiness of newly graduated nurses for practice generally shows that there are no significant concern about the readiness of newly graduated nurses for practice that is consistently supported by strong evidence. However, some studies have found that NGNs often report feeling unprepared for clinical practice due to a lack of self‐confidence (Ulupinar and Aydogan 2021; Najafi and Nasiri 2023; Masso et al. 2022), with many indicating poor performance in patient care (Maria et al. 2020; Kim and Shin 2022). The insufficient work readiness of NGNs may contribute to an increased frequency of adverse events in healthcare settings, delays in patient recovery, and negative impacts on patient safety (Brown et al. 2022; Kim et al. 2024).

Another key concept explored in this study is vocational belonging. Maslow (1987) states that a good society must meet the needs of individuals in order to survive and thrive. The first two levels of Maslow's hierarchy of needs consist of physiological needs such as eating, drinking, and breathing, which must be met in order to survive, and the need for safety, which is necessary to continue living in security. The third level is the need for belonging (Akdeniz and Tekeli 2024). Maslow (1968) placed the sense of belonging in the middle of the motivational hierarchy pyramid, attaching great importance to the sense of belonging and social acceptance throughout his life. The primary interest of belonging need is a psychological sense of being in a state of communion with others or a safe unit. Human beings need to live collectively or belong to a group that allows rooting and generates identity and social reference. Sense of belonging is the biggest reason to form groups, communities, and societies. All people feel the need to belong—to be part of something through identification. The fact that the need for belonging immediately follows the first two needs, which must be met as a biological necessity of human existence, indicates that it is an important step on the path to the top of the pyramid and serves to help the individual reach the highest level (Akdeniz and Tekeli 2024).

Mahar et al. (2013), who developed the concept of belongingness, explain this concept as the result of a mutual relationship developed on the basis of subjective, shared experiences, beliefs, or personal characteristics. These feelings of external connectedness are grounded to the context or referent group, to whom one chooses, wants and feels permission to belong. This dynamic phenomenon may be either hindered or promoted by complex interactions between environmental and personal factors.

Among the fundamental elements that contribute to an individual's sense of safety is the feeling of belonging, which may be directed toward a person, group, family, profession, society, culture, or place. In a professional context, vocational belonging refers to an individual's engagement with their occupation, their identification with it, awareness of their professional responsibilities, commitment to maintaining their career, and the emotional connection they feel toward their profession (Pamuk Cebeci and Uslu 2024).

The more an individual embraces the values and goals of their work organisation and institution, or the more they feel a sense of belonging to their work organisation and institution, the more willing they are to perform their duties for the benefit of that organisation and institution. An employee who does not feel a sense of belonging to their profession and the institution where they work, or more explicitly, who is dissatisfied with their career choice or the attitudes of their managers and colleagues, whose social and economic needs are not met, who does not feel at home in their workplace, and whose demands are not met, cannot be productive and successful in their profession. However, the degree of professional belonging varies from person to person. Individuals with high professional identity are more willing to identify themselves with their profession and institution, participate in processes related to their profession and institution, make efforts on behalf of their profession and institution, and continue their profession. In this case, it is inevitable for institutions to prefer employees with high professional identity (Benner 1984; Keskin and Pakdemirli 2016).

A strong sense of vocational belonging is particularly important in the nursing profession, which is human‐centered and values the uniqueness and well‐being of individuals (Pamuk Cebeci and Uslu 2024). When nurses experience a lack of vocational belonging, it may lead to dissatisfaction and challenges in both their personal and professional lives. This can adversely affect the quality of patient care, contribute to emotional distress such as depressive symptoms, and ultimately harm nurse–patient relationships (Ozdemir et al. 2022; Pamuk Cebeci and Uslu 2024). Kox et al. (2020), in their study of newly employed nurses in the Netherlands, found that a perceived lack of belonging was a key factor contributing to early career turnover (Punt 2022). In contrast, a strong sense of belonging, particularly during the early years of practice (Middleton et al. 2021), has been associated with greater job satisfaction and retention, positioning it as a critical factor in achieving sustainable professional practice (Biles et al. 2024). Building vocational belonging through professional competence may therefore serve as a protective factor against early attrition in the nursing workforce (Punt 2022). Despite its importance, few studies have investigated the relationship between work readiness and vocational belonging among NGNs. Further research is needed to explore how NGNs perceive their preparedness for professional practice and the extent to which they feel connected to their chosen field. In this context, the present study is considered significant for addressing this gap in the literature by examining the following research questions:

1. Are NGNs prepared for work?
2. Do NGNs feel a sense of belonging to the profession?
3. Is there a relationship between the levels of work readiness and vocational belonging among NGNs?
4. Does the work readiness of NGNs influence their level of vocational belonging?

## 3 Methodology

### 3.1 Design and Sample

The study employed a descriptive, correlational, and cross‐sectional design. The study was reported in accordance with the STROBE checklist.

The research population consisted of 2000 NGNs employed at a city hospital in Istanbul, Türkiye (A tertiary care hospital converted into a Covid‐19 pandemic hospital). The required sample size was calculated using an online sample size calculator (https://clincalc.com/stats/samplesize.aspx). Based on a prior study by Yildiz Keskin and Aslan (2021), which reported a mean WRS‐NGN score of 364.77 ± 51.89, the minimum sample size was determined to be 364, with a 95% confidence interval and a 5% margin of error. To accommodate an anticipated 20% non‐response rate, the questionnaire was distributed to 436 NGNs. The final research sample consisted of 415 participants, selected using a convenience sampling method (response rate: 95.18%). A post hoc power analysis was conducted using the G*Power (3.1.9.2) program, which revealed an effect size (*f* ^2^) of 0.85, indicating a large effect, and a statistical power of 100% (1.00), confirming the adequacy of the sample size.

*Inclusion criteria were*:

1. holding at least a bachelor's degree in nursing,
2. willingness to participate, and
3. having less than 3 years of professional experience in nursing.
4. Graduation date from the nursing bachelor's program less than 3 years ago.

*Exclusion criteria included*:

1. declining participation, and
2. having more than 3 years of professional experience in nursing,
3. being employed in other healthcare roles.
4. Graduation date from the nursing bachelor's program more than 3 years ago.

### 3.2 Data Collection Tools

The following measurement tools were used in this study:

#### 3.2.1 Descriptive Information Form

This form was developed by the researchers based on a comprehensive review of the literature. It consists of five questions, including individual and professional characteristics (age, gender, marital status, education level, professional experience in nursing).

#### 3.2.2 The Work Readiness Scale‐Newly Graduated Nurses (WRS‐NGN)

The 64‐item Work Readiness Scale, originally developed by Caballero et al. (2011) to assess work readiness among graduates, was adapted for the nursing profession by Walker et al. (2015), reducing the scale to 46 items. The Turkish validity and reliability study was conducted by Yildiz Keskin and Aslan (2021). The scale evaluates 4 factors: work competence, social intelligence, organisational acumen, and personal work characteristics and rated on a 10‐point Likert scale, ranging from “Strognly disagree” to “Strongly agree.” Some items are reverse‐scored. The total scale score is obtained by summing all item scores, with possible scores ranging from 46 to 460, where higher scores indicate a greater work readiness among NGNs. In the study by Walker et al. (2015), the Cronbach's alpha value for the scale was determined to be 0.92, with the following factor values: work competence (0.88), social intelligence (0.87), organisational acumen (0.85), and personal work characteristics (0.84). In the study by Yildiz Keskin and Aslan (2021), the Cronbach's alpha reliability coefficient for the overall scale was found to be 0.94, with the factors showing the following values: work competence and social intelligence (0.91), organisational acumen (0.93), and personal work characteristics (0.88). In this research, the Cronbach's alpha coefficient for the overall scale was found to be 0.96. The reliability coefficients for the factors were as follows: work competence (0.94), social intelligence (0.94), organisational acumen (0.96), and personal work characteristics (0.92).

#### 3.2.3 The Vocational Belonging Scale (VBS)

Developed by Keskin and Pakdemirli (2016), the VBS scale consists of three factors and 39 items: vocational management belonging, vocational organisation belonging, and vocational place belonging. The scale employs a 5‐point Likert type format, with response options ranging from “Strongly disagree” to “Strongly agree.” The total score of the scale ranges from 39 to 195, where higher scores indicate a stronger sense of vocational belonging. In the original study by Keskin and Pakdemirli (2016), the Cronbach's alpha reliability coefficient for the overall scale was 0.95, with the following values for its factors: vocational management belonging (0.95), vocational organisation belonging (0.92), and vocational place belonging (0.76) (Keskin and Pakdemirli 2016). In the present study, the VBS demonstrated excellent internal consistency, with a Cronbach's alpha of 0.97 for the overall scale, with the following values for its factors: vocational management belonging (0.93), vocational organisation belonging (0.97), and vocational place belonging (0.83).

### 3.3 Data Collection

The researchers distributed the study questionnaires to volunteers between April 26, 2021, and May 20, 2021. Each questionnaire was provided in a sealed envelope, completed by the participants, and then collected by the researchers. The data collection process took approximately 5 to 10 min per participant.

### 3.4 Data Analysis

All data in this study were analysed using IBM SPSS Statistics version 22.0. Skewness and kurtosis values were examined to assess the normality of the data distribution. Descriptive statistical methods (such as frequency, percentage, arithmetic mean, standard deviation, and minimum and maximum values) were used to evaluate the participants' personal and occupational characteristics. Based on the assumption of normal distribution, parametric tests including the independent samples *t*‐test and ANOVA were applied for group comparisons. Pearson correlation analysis was used to assess relationships between variables. When significant differences were found among multiple groups, the Bonferroni correction was applied as a post hoc test to determine which groups differed significantly. In addition, multiple linear regression analysis was conducted to examine the predictive relationships between scale scores. Cronbach's alpha coefficient was used to assess the internal consistency and reliability of each scale. All results were interpreted using a 95% confidence interval, with a significance level set at *p* < 0.05.

### 3.5 Ethical Considerations

Ethics committee approval (Date: 12.04.2021/Issue: 53) and institutional permission were obtained from X Hospital to conduct the research. Prior to administering the scales, participants were informed about the purpose of the study, and verbal consent was obtained from each nurse. Written permissions for the use of the data collection tools were secured from the original authors via email. All procedures in this study were carried out in accordance with the principles of the Declaration of Helsinki.

## 4 Results

The individual and professional characteristics of NGNs are presented in Table 1.

**TABLE 1:** Distribution of participants according to individual and professional characteristics data (*n* = 415).

Variables	*N*	%
Age		
21–24	149	35.90
25–27	218	52.53
≤ 28	48	11.57
Gender		
Female	277	66.75
Male	138	33.25
Marital status		
Married	66	15.90
Single	349	84.10
Education level		
Bachelor's degree	391	94.20
Master	24	5.80
Professional experience in nursing		
< 1 year	224	54.00
1–2 year	81	19.50
2 > *x* ≥ 3 year	110	26.50

Variables	Minimum–Maximum	Mean	SD
Age	21–36	25.45	2.40
Professional experience in nursing	8 month–26 month	1.72	0.86

It was found that 52.53% of the participants were between 25 and 27 years of age, with an average age of 25.45 ± 2.40 years. Among the participants, 66.75% were female, 84.10% were single, and 94.20% held a bachelor's degree. The duration of professional experience in nursing ranged from 8 to 26 months, with 54.0% reporting less than one year of experience. The average duration of professional employment was 1.72 ± 0.86 years (Table 1).

Reliability measurements and mean scores for the WRS‐NGN and VBS, including their respective factors, are presented in Table 2.

**TABLE 2:** Distribution of reliability measures, mean scores, and subscales for WRS‐NGN and VBS (*n* = 415).

Scale and sub‐dimensions	Scala min–max values	Mean	SD	Item score mean	SD	Cronbach alpha
Work competence	14–140	105.36	20.60	7.53	1.47	0.94
Social intelligence	8–80	63.04	12.78	7.88	1.60	0.94
Organisational acumen	16–160	131.34	23.32	8.21	1.46	0.96
Personal work characteristics	8–80	47.53	18.08	5.94	2.26	0.92
WRS‐NGN	46–460	347.29	55.37	7.55	1.20	0.96
Vocational management belonging	18–90	61.57	15.60	3.42	0.87	0.93
Vocational organisation belonging	16–80	45.12	12.24	2.82	0.77	0.97
Vocational place belonging	5–25	15.13	5.22	3.03	0.33	0.83
VBS	39–195	121.81	35.45	3.12	0.91	0.97

Abbreviations: VBS, vocational belonging scale; WRS‐NGN: the work readiness scale‐newly graduated nurses.

The total mean score for the WRS‐NGN was 7.55 ± 1.20, and the factor means were as follows: work competence 7.53 ± 1.47, social intelligence 7.88 ± 1.60, organisational acumen 8.21 ± 1.46, and personal work characteristics 5.94 ± 2.26. The total mean score for VBS among NGNs was 3.12 ± 0.91. The factor means were as follows: vocational management belonging 3.42 ± 0.87, vocational organisation belonging 2.82 ± 0.77, and vocational place belonging 3.03 ± 0.33. The Cronbach's alpha reliability coefficients for the scales were 0.96 for WRS‐NGN, with its factors ranging from 0.92 to 0.96, and 0.97 for VBS, with the reliability coefficients for its factors ranging from 0.83 to 0.97 (Table 2).

The scores obtained by NGNs from the WRS‐NGN and VBS according to their personal and occupational characteristics are presented in Table 3.

**TABLE 3:** The relationship between WRS‐NGN and VBS and its sub‐dimensions in new graduate nurses according to their descriptive characteristics (*n* = 415).

Variables		WRS‐NGN		*t*/*F* test	VBS		*t*/*F* test
		Mean	SD		Mean	SD	
Age	21–24	342.61	52.74	*F* = 2.08 *p* = 0.13	115.12	37.65	*F* = 5.59 *p* < 0.001***
	25–27	347.39	57.63		123.90	33.28	
	≤ 28	361.31	51.42		133.10	34.67	
Gander	Famale	351.12	52.30	*t* = 2.01 *p* = 0.04*	122.55	35.27	*t* = 0.60 *p* = 0.55
	Male	339.59	60.54		120.33	35.90	
Marital status	Married	359.88	47.27	*t* = 2.02 *p* = 0.04*	128.71	32.44	*t* = 1.73 *p* = 0.08
	Single	344.91	56.52		120.51	35.89	
Education level	Bachelor's degree	358.50	55.81	*t* = 2.93 *p* = 0.03*	121.91	35.22	*t* = 0.74 *p* = 0.53
	Master	406.00	45.25		120.25	40.17	
Professional experience	< 1 year	337.75	55.64	*F* = 10.89 *p* < 0.01**	120.95	34.33	*F* = 2.21 *p* = 0.11
	1–2 year	346.73	58.91		116.78	36.59	
	2 > *x* ≥ 3 year	367.13	46.70		127.29	36.45	

*Note:* *t*, independent two‐group *t*‐test; *F*, ANOVA.

* *p* < 0.05.

** *p* < 0.01.

*** *p* < 0.001.

A significant difference was found in WRS‐NGN scores based on gender, marital status, educational level, and duration of professional experience (*p* < 0.05). However, no statistically significant difference was observed in work readiness scores across different age groups, although a trend of increasing scores with age was noted (F = 2.08, *p* = 0.13). The mean WRS‐NGN scores were 342.61 ± 52.74 for NGNs aged 21–24 years, 347.39 ± 57.63 for those aged 25–27 years, and 361.31 ± 51.42 for those aged over 28. In terms of gender, female NGNs had significantly higher work readiness scores (351.12 ± 52.30) compared to males (339.59 ± 60.54) (t = 2.01, *p* = 0.04). Marital status also showed a significant correlation with work readiness: married NGNs scored higher (359.88 ± 47.27) compared to single NGNs (344.91 ± 56.52) (t = 2.02, *p* = 0.04). Regarding educational attainment, NGNs with a master's degree scored significantly higher (406.00 ± 45.25) than those with a bachelor's degree (358.50 ± 55.81) (*t* = 2.93, *p* = 0.03). The duration of professional experience was positively correlated with work readiness. NGNs with 2–3 years of experience had the highest scores (367.13 ± 46.70), followed by those with 1–2 years (346.73 ± 58.91), and those with less than one year (337.75 ± 55.64) (*F* = 10.89, *p* < 0.01) (Table 3).

In relation to the VBS, a statistically significant difference was found across age groups (*p* < 0.01). Mean VBS scores were: 115.12 ± 37.65 for ages 21–24, 123.90 ± 33.28 for ages 25–27, and 133.10 ± 34.67 for those aged over 28 (F = 5.59, *p* < 0.01). Hovewer a difference was not found in VBS scores based on gender, marital status, educational level, and duration of professional experience (*p* < 0.05) (Table 3).

The relationship between NGNs' WRS‐NGN and VBS and their subscales with personal and occupational characteristics is presented in Table 4.

**TABLE 4:** The relationship between of NGNs' WRS‐NGN and VBS and its sub‐dimensions whit their personal and occupational characteristics (*n* = 415).

Scale and sub‐dimensions		Test	Gender	Marital status	Education level	Test	Age	Professional experience
WRS‐NGN		*t*	2.01	2.02	2.93	*F*	2.08	10.89
		*p*	0.04*	0.04*	0.03*	*p*	0.13	*p* < 0.001***
WRS‐NGN sub‐dimensions	Work competence	*t*	−0.04	2.53	2.53	*F*	6.23	20.63
		*p*	0.96	0.06	0.06	*p*	*p* < 0.001***	*p* < 0.001***
	Social intelligence	*t*	1.42	2.08	2.08	*F*	1.72	11.59
		*p*	0.16	0.10	0.10	*p*	0.18	*p* < 0.001***
	Organisational acumen	*t*	2.60	1.93	1.93	*F*	0.50	3.59
		*p*	*p* < 0.001**	0.12	0.12	*p*	0.61	0.03*
	Personal work characteristics	*t*	1.85	1.53	1.53	*F*	0.26	1.98
		*p*	0.06	0.21	0.21	*p*	0.77	0.14
VBS		*t*	0.60	1.73	0.74	*F*	5.59	2.21
		*p*	0.55	0.08	0.53	*p*	*p* < 0.001***	0.11
VBS sub‐dimensions	Vocational organisation belonging	*t*	0.67	0.31	0.31	*F*	4.18	1.48
		*p*	0.51	0.82	0.82	*p*	0.02*	0.23
	Vocational place belonging	*t*	0.35	0.6	0.6	*F*	4.33	2.41
		*p*	0.73	0.61	0.61	*p*	0.01*	0.09
	Vocational management belonging	*t*	0.49	1.16	1.16	*F*	5.53	3.04
		*p*	0.63	0.32	0.32	*p*	*p* < 0.001***	0.04*

Abbreviations: VBS, vocational belonging scale; WRS‐NGN, the work readiness scale‐newly graduated nurses.

* *p* < 0.05.

** *p* < 0.01.

*** *p* < 0.001.

A significant difference was observed in the organisational acumen factor of the WRS‐NGN in relation to gender (*t* = 2.60, *p* < 0.05). In addition, significant differences were identified in overall work readiness based on age (*F* = 6.23, *p* < 0.01) and duration of professional experience (*F* = 20.63, *p* < 0.01). The social intelligence factor also showed a statistically significant difference based on the duration of professional experience (*F* = 11.59, *p* < 0.01). Furthermore, organisational acumen differed significantly not only by gender (*t* = 2.60, *p* < 0.01) but also by duration of professional experience (*F* = 3.59, *p* = 0.03) (Table 4).

A statistically significant difference was found between all factors of the VBS and the age of the participants (*p* < 0.05). In addition, a significant difference was observed between vocational management belonging and duration of professional experience (*F* = 3.04, *p* = 0.04). A significant difference was also found in the total VBS score based on age (*F* = 4.04, *p* = 0.02), as well as in the factors of vocational place belonging (*F* = 4.33, *p* = 0.01) and vocational management belonging (*F* = 5.59, *p* < 0.01) (Table 4).

The correlation analysis between the WRS‐NGN and VBS, along with their respective factors, is presented in Table 5.

**TABLE 5:** The relationship between Newly graduated nurses' WRS‐NGN and VBS (*n* = 415).

	WRS‐NGN	Work competence	Social intelligence	Organisational acumen	Personal work characteristics	VBS	Vocational organisation belonging	Vocational place belonging	Vocational management belonging
WRS‐NGN									
*r*	1								
*p*									
*N*	415								
Work competence	0.866**	1							
	*p* < 0.01								
	415	415							
Social intelligence									
*r*	0.850***	0.807***	1						
*p*	*p* < 0.01	*p* < 0.01							
*N*	415	415	415						
Organisational acumen	0.871***	0.721***	0.736***	1					
	*p* < 0.001	*p* < 0.001	*p* < 0.001						
	415	415	415	415					
Personal work characteristics									
*r*	0.351***	0.010	0.027	0.035	1				
*p*	*p* < 0.001	0.837	0.580	0.476					
*N*	415	415	415	415	415				
VBS									
*r*	0.607***	0.541***	0.570***	0.654***	−0.086	1			
*p*	*p* < 0.001	*p* < 0.001	*p* < 0.001	*p* < 0.001	0.079				
*N*	415	415	415	415	415	415			
Vocational organisation belonging	0.577***	0.238***	0.364***	0.420***	−0.129**	0.931**	1		
	*p* < 0.001	*p* < 0.001	0.001	*p* < 0.001	0.008	*p* < 0.01			
	415	415	415	415	415	415	415		
Vocational place belonging									
*r*	0.675***	0.395***	0.320***	0.507***	−0.045	0.915**	0.836***	1	
*p*	*p* < 0.001	*p* < 0.001	*p* < 0.001	*p* < 0.001	0.356	*p* < 0.01	*p* < 0.001		
*N*	415	415	415	415	415	415	415	415	
Vocational management belonging									
*r*	0.512***	0.405***	0.354***	0.452***	−0.034	0.907***	0.698***	0.795***	1
*p*	*p* < 0.001	*p* < 0.001	*p* < 0.001	*p* < 0.001	0.495	*p* < 0.01	*p* < 0.001	*p* < 0.001	
*N*	415	415	415	415	415	415	415	415	415

Abbreviations: VBS, vocational belonging scale; WRS‐NGN, the work readiness scale‐newly graduated nurses.

** *p* < 0.01.

*** *p* < 0.001.

A moderate, positive, and statistically significant correlation was found between the total scores of WRS‐NGN and VBS (*r* = 0.607, *p* < 0.01). Within the WRS‐NGN, very strong positive correlations were observed between the total scale score and its factors: work competence (*r* = 0.866, *p* < 0.01), social intelligence (*r* = 0.850, *p* < 0.01) and organisational acumen (*r* = 0.871, *p* < 0.01). A moderate positive correlation was found with the personal work characteristics factor (*r* = 0.351, *p* < 0.01). Regarding the VBS, very high, positive, and significant correlations were found between the total scale and its factors: vocational management belonging (*r* = 0.907, *p* < 0.01), vocational place belonging (*r* = 0.915, *p* < 0.01), and vocational organisation belonging (*r* = 0.907, *p* < 0.01). Furthermore, moderate, positive, and statistically significant correlations were found between the total WRS‐NGN score and the factors of VBS: vocational organisation belonging (*r* = 0.577, *p* < 0.01), vocational place belonging (*r* = 0.675, *p* < 0.01), and vocational management belonging (*r* = 0.512, *p* < 0.01). Similarly, moderate, positive correlations were found between the total VBS score and the WRS‐NGN factors of work competence (*r* = 0.541, *p* < 0.01), social intelligence (*r* = 0.570, *p* < 0.01), and organisational acumen (*r* = 0.654, *p* < 0.01) (*p* < 0.01). However, no statistically significant correlation was found between the total VBS score and the personal work characteristics factor of WRS‐NGN (r = −0.86, *p* = 0.079) (*p* > 0.05) (Table 5).

The results of the multiple regression analysis‐Model 1 conducted to examine the predictive effect of demographic variables on job readiness and The results of the multiple regression analysis Model 2 conducted to examine the predictive effect of the WRS‐NGN factors on the factors of VBS are presented in Table 6.

**TABLE 6:** The results of the multiple regression analysis (*n* = 415).

Model 1							
WRS‐NGN and variables	*B*	Std. error	*β*	*p*	*t*	95% CI	
						Lower bound	Upper bound
Constant	333.088	44.587		*p* < 0.001**	7.471	245.439	420.736
Age	−0.072	1.224	−0.003	0.953	−0.058	−2.478	2.335
Gender	−6.763	5.562	−0.06	0.225	−1.216	−17.697	4.171
Marital status	−6.44	7.634	−0.044	0.399	−0.844	−21.448	8.568
Education level	4.681	9.616	0.024	0.627	0.487	−14.222	23.584
Professional experience	13.958	3.225	0.223	*p* < 0.001**	4.329	7.619	20.298
*R* = 0.248; *R* ^2^ = 0.62; *F* = 5.453; Adjusted *R* ^2^ = 0.50; *p* < 0.001** Durbin‐Watson:1.640.							

Model 2				
WRS‐NGN and sub‐dimensions	Vocational organisation belonging	Vocational place belonging	Vocational management belonging	VBS
Work competence				
*β*	0.420	0.495	0.408	0.426
*t*	4.88	6.286	2.674	3.025
*p*	*p* < 0.001**	*p* < 0.001**	0.008**	0.003**
Social intelligence				
*β*	0.386	0.320	−0.179	−0.227
*t*	3.330	4.594	−0.994	−1.532
*p*	0.001**	*p* < 0.001**	0.321	0.126
Organisational acumen				
*β*	0.317	0.407	0.462	0.472
*t*	4.516	6.560	5.326	3.847
*p*	*p* < 0.001**	*p* < 0.001**	*p* < 0.001**	*p* < 0.001**
Personal work characteristics				
*β*	−0.336	−0.045	−0.046	−0.198
*t*	−2.783	−0.924	−1.060	−2.163
*p*	0.006**	0.356	0.290	0.031*
Constant	*R* = 0.582, *R* ^2^ = 0.339, *F* = 9.511, Adjusted *R* ^2^ = 0.332 *p* < 0.001**	*R* = 0.684, *R* ^2^ = 0.468 *F* = 13.494, Adjusted *R* ^2^ = 0.463 *p* < 0.001**	*R* = 0.564, *R* ^2^ = 0.309, *F* = 29.117. Adjusted *R* ^2^ = 0.301 *p* < 0.001**	*R* = 0.588. *R* ^2^ = 0.346. *F* = 18.609. Adjusted *R* ^2^ = 0.341 *p* < 0.001**
WRS‐NGN	*B* = 289.127; *R* = 0.607; *R* ^2^ = 0.368; *t* = 6.556; SE = 52.76; *p* < 0.001**			

Abbreviations: *β*, Standardised coefficient; *B*, unstandardised coefficient.

* Correlation is significant at the 0.05 level (2‐tailed).

** Correlation is significant at the 0.01 level (2‐tailed).

In the multiple regression model, the professional experience explains only 5% (*R* = 0.248; *R* ^2^ = 0.62; *F* = 5.453; Adjusted *R* ^2^ = 0.50; *p* < 0.001) of the perceived readiness for work among the specified demographic criteria. It was determined that this value was statistically significant but provides a very low level of contribution (Table 6).

The dependent variable in the analysis was the vocational belonging scores of the nurses. The findings revealed that the work competence, social intelligence, organisational acumen, and personal work characteristics factors of WRS‐NGN had a statistically significant effect on the vocational organisation belonging factor of VBS (*R* = 0.582, *R* ^2^ = 0.339, Adjusted *R* ^2^ = 0.332, *F* = 9.511, *p* < 0.01), explaining 33.2% of the observed variance. In terms of vocational place belonging, the factors of work competence, social intelligence, and organisational acumen were found to be significant predictors (*R* = 0.684, *R* ^2^ = 0.468, Adjusted *R* ^2^ = 0.463, *F* = 13.494, *p* < 0.01), accounting for 46.3% of the variance. For the vocational management belonging factor, work competence and organisational acumen were identified as significant contributors (*R* = 0.564, *R* ^2^ = 0.309, Adjusted *R* ^2^ = 0.301, *F* = 29.117, *p* < 0.01), explaining 30.1% of the variance. When evaluating the total vocational belonging score, the factors of work competence, organisational acumen, and personal work characteristics were found to significantly predict the outcome (*R* = 0.588, *R* ^2^ = 0.346, Adjusted *R* ^2^ = 0.341, *F* = 18.609, *p* < 0.01), explaining 34.1% of the observed variance. In the model established in the study, 36.8% of the variance in vocational organisation belonging was explained by work readiness (*R* = 0.607, *R* ^2^ = 0.368, *t* = 6.556, *p* < 0.01) (Table 6).

## 5 Discussion

The work readiness and vocational belonging of NGNs upon entering the workforce remains an important area of discussion. This study examined the relationship between NGNs' work readiness and their level of vocational belonging.

The findings indicated that NGNs' overall work readiness, as measured by the WRS‐NGN scale, was at a good level. Among four factors, the highest mean score was observed in the organisational acumen factor, while the lowest score was reported in personal work characteristics (Table 2). Considering that many participants completed their education and entered professional practice during the COVID‐19 pandemic, these results suggest a relatively high level of perceived readiness. This finding aligns with results from Yildiz Keskin and Aslan (2021), where personal work characteristics similarly received the lowest scores. Comparable outcomes have also been reported by Gao et al. (2022), Ma et al. (2023), and Li et al. (2022). In contrast, a study conducted in Australia using the same scale found higher levels of work readiness among NGNs who entered the profession during the pandemic (Qu et al. 2025). On the other hand, NGNs in Türkiye were found to exhibit moderate levels of work readiness (Tarhan et al. 2022), highlighting the need for further development in this area. The many educational institutions struggle to meet these requirements, and the lack of standardisation in nursing education across the country remains a challenge (Ulupinar and Aydogan 2021; Chen et al. 2021).

A noteworthy finding is that the lowest score among the sub‐dimensions of the WRS‐NGN was obtained in the personal work characteristics dimension. However, the lower scores in personal work characteristics indicate that NGNs may experience deficiencies in psychological or intrapersonal attributes relevant to professional development (Ma et al. 2023). Personal work characteristics dimension; it is emphasised that nurses should have some personal characteristics in order to be successful in their profession (Walker et al. 2015). Especially for newly graduated nurses, the development of these characteristics is vital for professional adaptation and resilience. These competencies are communication and personal skills, empathy and compassion, realism and flexibility, honesty and reliability, and time management skills. This set of personal competencies directly affects the capacity of new graduate nurses to manage professional stress and establish a healthy balance between professional and personal life, thereby reducing the risk of burnout syndrome and supporting long‐term career success (Yildiz Keskin and Aslan 2021). In this regard, it is evident that the personal work characteristics of newly graduated nurses need to be improved in order for them to be ready for work.

Studies show that NGNs graduate without sufficient clinical experience, and their training often fails to reflect the realities of modern nursing practice. As a result, they may lack the professional knowledge and skills required for effective care delivery (Ulupinar and Aydogan 2021; Chen et al. 2021). Evidence further suggests that professional programs and opportunities to enhance clinical competence significantly contribute to job satisfaction and reduce early career turnover among NGNs (Charette et al. 2020).

Another key finding of this study is that NGNs demonstrated a moderate level of vocational belonging, indicating that this area required further reinforcement to foster a stronger sense of professional identity and commitment. On the other hand, it was also found that more than half of the NGNs reported a high level of vocational belonging. This dual finding suggest variability among NGNs in terms of their sense of vocational belonging. The present results are supported by studies such as those by ElSadany et al. (2024) and Taylor et al. (2022), which similarly report that a considerable proportion of NGNs exhibit a strong sense of vocational belonging. This may be attributed to supportive environments within healthcare organisations, where leadership and management practices promote a sense of inclusion and ownership among NGNs. However, it is important to note that nearly half of the participants indicated a level of vocational belonging that falls below the desired threshold. In contrast to the current findings, Punt (2022) reported that most nurses exhibited low levels of vocational belonging. In addition, other studies have identified widespread dissatisfaction among nurses, including negative attitudes toward their roles (Ching et al. 2022) and their healthcare institutions (Fernando et al. 2022), which diverges from the more positive perceptions observed in this study.

This study is the first to examine the relationship between work readiness and vocational belonging, highlighting the positive influence that work readiness exerts on fostering a stronger sense of vocational belonging among NGNs. The findings revealed a significant positive correlation between levels of work readiness and vocational belonging. As NGNs' work readiness improves, their professional awareness and identity strengthen, resulting in a heightened sense of vocational belonging. The study also found that work readiness significantly predicts vocational belonging. This aligns with previous research indicating that work readiness is a predictor of vocational commitment (Li et al. 2022), which, in turn, has been shown to predict organisational commitment. Therefore, the effect of work readiness on vocational belonging may operate through both direct and indirect pathways, reflecting the multifaceted nature of this relationship. Overall, the results of this study emphasise that enhancing work readiness can play a critical role in promoting vocational belonging among NGNs. Being work ready allows individuals to adapt more easily to their professional roles, which, in turn, supports the development of their professional identity. In the specific context of nursing, professional identity has played a critical role in the profession's evolution into a respected position and in the adoption of ethical values. This identity development is a continuous process nourished by education and professional practices. As newly graduated nurses integrate into the workforce, their professional identity is further shaped. Nurses who feel competent and knowledgeable in their work assign a deeper meaning and stronger bond to their profession. This strong bond eventually transforms into a sense of vocational belonging (Bakhshi Zadeh et al. 2025). Consequently, as the level of job readiness increases, professional consciousness and, as a result, professional belonging also rise.

Work readiness emerges as a primary factor reinforcing an employee's organisational commitment. This effect manifests through the individual's enhanced perception of competence and success in their work. An employee's success and recognition in their job, stemming from their readiness, lay the groundwork for developing a stronger positive emotional bond with the organisation. This naturally increases affective commitment and fosters a strong sense of professional belonging. Furthermore, work readiness strengthens continuance commitment by decreasing the costs of leaving through gains such as internal promotions, higher salaries, and significant benefits (Hosen et al. 2024). This situation positively influences all three fundamental dimensions of commitment—affective, continuance, and normative commitment—as proposed by Meyer and Allen (1991), thereby reinforcing the individual's sense of professional belonging. This multi‐dimensional commitment forms the foundation for a sense of belonging to both the organisation and the profession. Consequently, as the level of job readiness rises, professional consciousness and, in turn, professional belonging also increase.

## 6 Conclusions

While NGNs in Türkiye exhibit a good level of work readiness, their vocational belonging remains at a moderate level, suggesting room for improvement. Given the significant role that work readiness plays in fostering vocational belonging, it is crucial for nurse educators and healthcare managers to design and implement effective transition strategies that support NGNs in achieving positive professional integration and development. Based on this results, it is concluded that merely equipping individuals with technical skills is insufficient. Creating socialisation environments that enable individuals to develop a sense of vocational belonging is crucial not only for personal career satisfaction and success but also for the formation of a more competent, motivated, and committed workforce at the societal level. Therefore, adopting a holistic approach in education and recruitment policies is a strategic necessity for ensuring sustainable workforce quality.

To this end, employers and/or managers can support newly graduated nurses by placing them in select teams and groups, creating environments where employees can communicate with each other, developing mentoring programs, and ensuring they are prepared for working life through well‐designed socialisation programs and psychological support programs. They can also cultivate an organisational culture that fosters a sense of belonging.

It was determined that readiness for work is an important predictor of perceived professional belonging among newly graduated nurses. It was concluded that professional competence gained through training newly graduated nurses to be ready for work and increasing their level of readiness through institutional orientation would increase their sense of professional belonging. Furthermore, the regression models in our study showed that although readiness for work accounted for one‐third of the total variance explained, it was largely determined by other factors. Therefore, to gain a more comprehensive understanding, future research should broaden its scope to explore the influence of relevant demographic, psychological, social, and organisational variables.

### 6.1 Limitations and Strengths

This study revealed the relationship between work readiness and vocational belonging among NGNs, thereby contributing significantly to the existing literature. However, certain limitations should be acknowledged. The data were collected through self‐reported responses from NGNs who voluntarily participated in the study, which introduces the potential for response bias and may limit the generalisability of the findings. The research was conducted within a single hospital setting, and transition programs for NGNs may vary across institutions, further constraining the applicability of the results to other contexts. Despite these limitations, the findings offer valuable insights and are expected to inform the efforts of nurse educators, nurse managers, and health policymakers in developing strategies to improve NGNs' work readiness and vocational belonging.

## 7 Relevance for Clinical Practice

Given the significant impact of work readiness on vocational belonging, the findings suggest that educators and healthcare managers should regularly assess the status of NGNs. It is crucial to recognise individual differences and offer tailored interventions and strategies to enhance their skills and vocational belonging, ensuring they are well‐prepared for the workforce. Health and educational institutions must prioritise initiatives aimed at improving the work‐life readiness of nursing students by strengthening collaboration and establishing a supportive organisational structure. Furthermore, NGNs should collaborate to explore methods for enhancing both their work readiness and vocational belonging, facilitating a smoother transition into the workforce. Educational programs should be continually evaluated and updated to reflect current knowledge, technology, and evidence‐based practices. In addition, providing sufficient clinical practice opportunities and supporting the transition to professional roles are crucial in helping NGNs improve their work readiness and vocational belonging.

Also there are several key implications for the management of NGNs based on the study's findings. First, it was hypothesised that NGNs with higher work readiness would exhibit a stronger sense of vocational belonging. Therefore, it is recommended that nurse educators assess work readiness and vocational belonging status prior to graduation. This evaluation would provide valuable insights into preparedness of NGNs for the workforce. In addition, hospital administrators could incorporate these assessments into their employment screening processes. These assessments could also inform decisions regarding transition programs, orientation sessions, and other support initiatives, ensuring that NGNs receive targeted assistance where needed. Secondly, identifying the predictors of work readiness and vocational belonging can help nursing managers recognise NGNs who may be less prepared for the workforce. This insight enables the implementation of more targeted continuing education programs designed to enhance work readiness and address any gaps in vocational belonging. NGNs are often eager to learn and excited to begin new careers, making them receptive to interventions that supports growth and professional development. By providing such support, nursing managers can enhance NGNs' work readiness while strengthening their sense of vocational belonging, which can lead to greater job satisfaction and retention.

## Author Contributions

**Berra Yilmaz Kusakli:** conceptualisation; investigation; writing – original draft; methodology; validation; visualisation; writing – review and editing; software; formal analysis; project administration; data curation; supervision; resources. **Ece Uysal Kasap:** conceptualisation; investigation; methodology; validation; writing – review and editing; software; formal analysis.

## Funding

The authors have nothing to report.

## Disclosure

Presentation statement: This study was presented as an oral presentation at the 1st National Nursing Management Congress held in Turkey on May 27–29, 2021.

## Ethics Statement

The study was approved by the Istanbul Basaksehir Cam and Sakura City Hospital Ethics Committee on April 12, 2021 (Approval Number: 53).

## Conflicts of Interest

The authors declare no conflicts of interest.

## Data Availability

The data that support the findings of this study are available from the corresponding author upon reasonable request.
